# Preparation of Azithromycin Nanofibers as Controlled Release Ophthalmic Drug Carriers Using Electrospinning Technique: *In Vitro* and *In Vivo* Characterization

**DOI:** 10.34172/apb.2022.033

**Published:** 2021-05-29

**Authors:** Shiva Taghe, Saba Mehrandish, Shahla Mirzaeei

**Affiliations:** ^1^Pharmaceutical Sciences Research Center, Health Institute, Kermanshah University of Medical Sciences, Kermanshah, Iran.; ^2^Nano Drug Delivery Research Center, Health Technology Institute, Kermanshah University of Medical Sciences, Kermanshah, Iran.; ^3^Research and Development Department, Rahesh Daru Novin Inc., Kermanshah University of Medical Sciences, Kermanshah, Iran.

**Keywords:** Azithromycin, Chitosan, Controlled release, Microbial assay, Nanofibers, Ophthalmic drug delivery

## Abstract

***Purpose:*** Conventional topical dosage forms face with some challenges like low intraocularbioavailability, which could be overcome by application of novel drug delivery systems.Therefore, this study was conducted to prepare azithromycin (AZM)-loaded chitosan/polyvinylalcohol/polyvinyl pyrrolidone (CS/PVA-PVP) nanofibers with the prolonged antibacterialactivity by electrospinning method.

***Methods:*** After preparation of nanofibers, they were characterized in terms of physicochemicaland morphological properties. *In vitro* and *in vivo* release of the drug from nanofibers wereevaluated using microbial assay against the *Micrococcus luteus*. Antibacterial efficacy of thenanofibers was assessed. The ophthalmic irritation test was also performed. MTT test wascarried out to evaluate cytotoxicity of the formulations.

***Results:*** All the formulations were found to be stable with uniform thickness, weight, and drugcontent. Nanofibers had a diameter range from 119 ± 29 to 171 ± 39 nm. The inserts were nonirritantand non-toxic to the rabbits’ eye. Based on the obtained results, the crosslinked AZMnanofibers showed slower and more controlled drug release in tear fluid compared to the noncrosslinkedones, within 184 hours.

***Conclusion:*** Our results revealed that the prepared nanofibers could be considered as suitableand non-invasive inserts for the prolonged ophthalmic delivery of AZM.

## Introduction

Topical dosage forms have always been the most common route of administration for ophthalmic anti-infective agents.^1^ The advantages of topical ophthalmic drug delivery include avoiding the first-pass metabolism, non-invasiveness, targeted delivery, high patient compliance, painlessness, and reduced side effects in comparison with systemic forms making it one of the most effective and popular routes of administration.^2,3^ Topical forms, especially eye drops are commonly used to treat ophthalmic diseases associated with anterior chamber. However, topical drug delivery has always faced with challenges, such as pre-corneal barriers including lacrimal drainage, blinking, tear film, and anatomical barriers reducing intraocular bioavailability of drug at the site of action. Given all these corneal–epithelial barriers, only 1-7% of the drug reaches aqueous humour and intra-ophthalmic tissues.^2,4^ As a result, novel drug delivery systems like hydrogels,^5^ nanofibers,^6^ liposomes,^7^ nanoemulsions,^8^ nanoparticles,^9,10^ etc., have been designed and developed to overcome these challenges. In the meantime, high-potential nano-based drug delivery systems have been developed including nanofibers with the increase in the use of nanotechnology in the industries.^11^ Electrospinning has always been the most popular technique for preparing the nanofibers.^12^ These systems have many advantages like high surface-area-to-volume ratio and high porosity; leading to high ability of the nanofibers to increase solubility, helping the controlled release of drug, and increasing intraocular bioavailability of drugs.^13,14^

Based on the previous studies, the cornea and conjunctiva have a negatively charged structure. Hence, positively charged mucoadhesive polymeric carriers interacting with the cornea and conjunctiva could increase contact time and consequently, concentration of drugs in the cornea.^15,16^

Chitosan (CS) is a cationic polymer with many beneficial properties, such as biodegradability, low toxicity, and biocompatibility making it one of the most popular polymers in design and preparation of the drug carriers.^17-20^ However, the nanofibers could be more beneficial by addition of other polymers like polyvinyl alcohol (PVA), polyvinyl pyrrolidone (PVP), and polyethylene terephthalate (PET) to CS compared to pure CS nanofibers.^21-23^ Also, this addition could lead to a significant decrease in fiber diameter of electrospun CS/PVP mixed fibers attributing to an increase in solution conductivity.^24,25^

Glutaraldehyde (GA) is a strong crosslinker due to high activity of aldehyde groups, which could interact with amino groups of proteins. Also, it can be used as a crosslinking agent for PVA and CS.^26,27^

In this study, azithromycin (AZM), as an effective agent against the external ophthalmic infections acting by inhibiting bacterial protein synthesis was loaded in the nanofibers composed of PVA, PVP, and CS blends and GA vapor-crosslinked nanofibers. After optimization and characterization of physicochemical properties, *in vitro* and *in vivo* release of the drug from the optimized nanofibers were evaluated. It was expected that the nanofibers achieve a controlled release of drug, which could decrease frequency of drug administration compared to conventional eye drops, and consequently increase the patient compliance.^28,29^

## Materials and Methods

### Materials

CS (70-58% deacetylated) was obtained from Acros Organics Company. PVA (99% hydrolyzed, average Mw = 89-98 kDa) and PVP were purchased from Aldrich Chemical Company. Acetic acid 100% was obtained from Merck Company (Darmstadt, Germany). All the other chemicals were of the best available grade.

### Preparation of polymer solutions

One percent (w/v) PVA and 3% (w/v) PVP solutions in distilled water were prepared at 50°C and were mixed for FZ-P formulation. Three percent (w/v) PVA and 1% (w/v) PVP solutions were prepared and were mixed for FZ-A formulation. For obtaining 2% (w/v) CS solution, CS was dissolved in acetic acid (1% v/v) at 25°C. CS/PVA-PVP solutions were obtained by addition of 20ml of CS solution to 20ml of PVA-PVP solutions with different polymer concentrations containing AZM (10% w/w of polymers) under continuous stirring at room temperature for 12 hours.

### Preparation of AZM nanofibers

**Figure 1:**
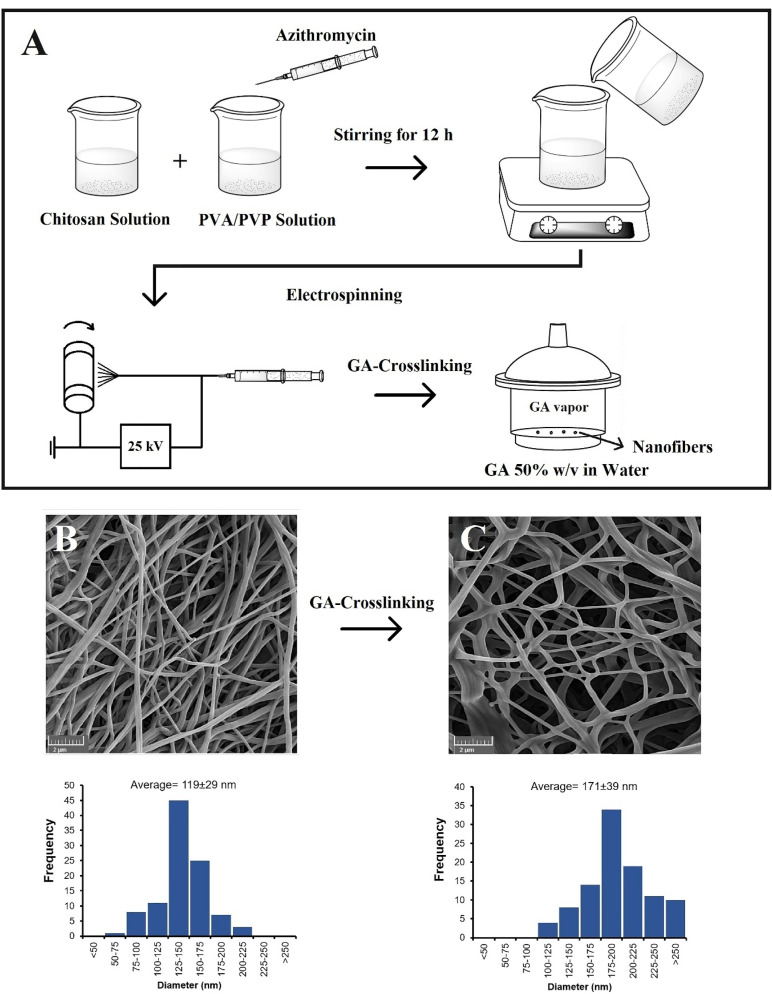
The experimental procedure for preparation of the nanofibers (A) along with SEM micrographs and histogram of diameter distribution corresponding to the non-crosslinked (B) and GA-crosslinked (C) electrospun nanofibers.

AZM nanofibers were prepared using a customized electrospinning system (Fanavaran Nano-Meghyas, Tehran). High voltage supply of 25 kV was applied to a stainless-steel capillary connected to a reservoir filled with polymeric solutions. Cylindrical collector was covered by a cellulose ester membrane to collect the electrospun samples. The whole electrospinning procedure was performed at 40°C. The experimental flow sheet is shown in Figure 1.

Crosslinking process was performed based on the method reported by Zhou et al.^30^ For preparing FZ-PG and FZ-AG formulations, FZ-P and FZ-A nanofibers were placed on a holed ceramic shelf in a desiccator containing 50%, v/v aqueous GA solution. For removing unreacted residual vapor-phase GA on the nanofibers, the formulations were initially soaked in 0.02 M glycine solution for 4 hours and then, in deionized water for 30 minutes. Finally, the fibers were dried under vacuum and were kept in a desiccator until further application.

### Physicochemical characterization of AZM nanofibers

#### Thickness, weight variation, and content uniformity

Thickness of the nanofibers was measured using digital micrometer ( ± 0.001 mm) in five different positions of nanofibers and an average was taken.

Weight variation in the nanofibers (6 mm of diameter) was tested using a digital weight balance (Shimadzu, Japan). Mean weight (n = 10) was recorded for each formulation.

For observing uniformity of the nanofibers’ drug content, same pieces of ophthalmic inserts were cut and dissolved into a specific amount of acetic acid (1% v/v), then were diluted with phosphate-buffered saline (PBS) solution and were filtered by filter papers (0.45 μm). The drug content was measured using microbiological assays.

#### Folding endurance

Nanofibers were cut into pieces with the same size, and then were folded at the same point repeatedly until they were torn. The number of times, in which the nanofibers could be folded without tearing or breaking, was reported as folding endurance.

#### Moisture uptake and loss

For estimating percentage of moisture uptake and loss, nanofibers were weighed initially and then, were placed respectively in a desiccator containing a saturated solution of aluminium chloride (to keep 79.5% of humidity inside the desiccator), and anhydrous calcium chloride for 72 hours. After that, the nanofibers were reweighed and percentage of moisture uptake and loss was calculated.^31^

#### Swelling percentage

The samples with initial weight of W_0_ were soaked in double-distilled water for 1 hour. After removing of the water from surface of nanofibers using a paper filter, they were reweighed to obtain W_t_. Swelling was calculated using the standard formula.

#### Determination of surface pH

The ophthalmic insert was allowed to swell in distilled water inside a Petri dish. The pH paper was placed on surface and after 1 minute; the developed color was compared with the standard color scale to estimate surface pH.

### Scanning electron microscopy (SEM)

Morphology of the nanofibers was observed using field-emission scanning electron microscopy (FE-SEM, MIRA3, TESCAN). The dried samples were placed on metal stubs with adhesive tape, were sputter-coated with gold and then, were observed under a scanning electron microscope.

### Fourier -transform infrared spectroscopy (FT-IR)

Samples were dried in a vacuum desiccator, were mixed with micronized KBr powder, and were compressed into a manual tablet press. Then, the FT-IR spectra were detected using (Shimadzu IR PRESTIGE-21, Japan) FT-IR spectrometer.

### Antimicrobial efficacy test

Specific amount of bacterial suspension was uniformly spread onto an agar plate. The nanofibrous mats were placed on the agar plates and were incubated at 35°C.^32^

Based on diameter of inhibition zones, against the Gram-positive organism *Staphylococcus aureus* (ATCC 6538) and the Gram-negative organism *Escherichia coli* (ATCC 9637), bactericidal effect of the nanofibers containing AZM was evaluated.

### Sterility test

For confirming sterility of the inserts, they were placed in thioglycollate broth, Sabouraud dextrose broth, and soybean-casein-digest broth, respectively to detect growth of aerobic bacteria, fungi, and anaerobic bacteria. The positive and negative controls were also prepared for comparison.

### Microbial assay

Microbial assay was performed using the *Micrococcus luteus*(ATCC 4698) by the standard disc diffusion method. The spread-plate technique was used^33^ and the culture media were incubated at 35°C for 24 hours. Bacterial suspension was prepared in PBS solution (pH = 7.4). The approximate number of bacteria in the suspension was standardized by comparing with turbidity of McFarland Standards. Then, the resultant bacterial suspension was used as culturing inoculum. The bacterial suspension was spread uniformly on a nutrient agar plate before placement of the disks. Sterile paper disks containing 30 μL of the samples were placed on the plates and then, were incubated at 35°C for 24 hours. Finally, mean diameter of the measured inhibition zones surrounding the paper disks was recorded in mm using Vernier caliper.

### In vitro study of drug release

For measuring the amount of the released drug from the nanofibers, specific amounts of the nanofibers were placed into donor compartments containing 1 mL of phosphate buffer at (pH 7.4) separated by a dialysis membrane (Delchimica Scientific Glassware, Milan, Italy), from the receptor compartments filled with 19 mL of the phosphate buffer. The system was stirred magnetically with the speed rate of 100 rpm at temperature of 37°C. Aliquots were withdrawn and replaced with the same amount of fresh buffer at specific time intervals to maintain the sink condition and the drug concentrations of the samples were quantified using the microbial assays.

### Irritation test

Adult New Zealand rabbits were used for assessment of ophthalmic tolerance. The animals were housed individually in restraining boxes and had free access to the allowed amounts of food and water. Sterile optimized formulations were administrated to one eye of the rabbits, while the other eye remained untreated without any manipulation as a reference. The eyes were observed in specific periods of time for damage, abnormality, swelling, redness, and inflammation as signs of irritancy.

### In vitro cytotoxicity test

L929 (mouse fibroblast) cells were cultured to evaluate cytotoxicity of the nanofibers. The 24-well plate was incubated for 48 hours. One row of 24-well plate that did not receive any formulations was considered as a control. Different concentrations of AZM-loaded nanofibers were administered to other rows followed by addition of 30 µL of MTT assay solution and 270 mL of medium to the wells. The plates were incubated for 4 hours. The precipitate was left in the wells after removing, and the solution was diluted with 150 µL of dimethyl sulfoxide (DMSO) solution and then, was observed using a microplate reader (GENios, Groedig, Austria) to estimate cell viability at the wavelength of 560 nm.

### In vivo study of drug release

Healthy rabbits were used to perform *in vivo* ocular studies. Eight rabbits were chosen as the experimental group while two of them were selected as the control group. In the experimental group, a bunch of nanofibers was placed in ocular sac of the rabbits. Sampling was done by pouring 50 μL of sterile phosphate buffer into ocular sac to dilute tear, which was collected by a paper disk. The paper disks containing tear samples were placed directly on a culture medium inoculated with standard bacterial suspension to estimate the amount of released drug in tear by the microbial assay method.

## Results and Discussion

### Preparation and evaluation of ophthalmic inserts of AZM-loaded nanofibers

AZM-loaded nanofibers with different formulations were successfully prepared using the electrospinning method. PVA was selected because it strongly interacts with CS through hydrogen bonding molecularly and it can be easily electrospun from aqueous solutions.^34^ PVP has many beneficial properties including high hydrophilicity, biocompatibility, ability to form the complex, and also fiber-forming ability.^35^ Addition of PVA/PVP to CS decreases viscosity and increases conductivity of the CS solution leading to the decreased diameter of the finally prepared nanofibers.^35^ This high conductivity suppresses varicose instability and enhances whipping instability causing formation of ultrafine fibers.^36^ Procedure of GA treatment was performed to crosslink the fiber mats using the Schiff base imine functionality to prepare insoluble nanofibers.^27^ After GA crosslinking, the nanofibers became slightly yellowish and their dimensions were decreased. The change in color occurred due to bonding of the free amine groups in the CS and PVA structures with GA. Drug loading was found to be uniform around 100% for all the formulations. Higher drug loading leads to a smaller insert size improving the patient compliance for application of AZM-loaded nanofibers. The prepared AZM-loaded nanofibers were characterized based on their physicochemical properties, such as moisture loss and uptake, thickness, degree of swelling, tensile strength, and folding endurance (Table 1). The prepared AZM-loaded nanofibers (6 mm of diameter) were observed to possess uniform weight and thickness (0.108 ± 0.012 to 0.121 ± 0.002 mm). As the prepared nanofibers are thin, they will not cause any ophthalmic irritation after placement in the eye tissues. The nanofibers indicated good folding endurance (153 ± 3 - 204 ± 3), revealing that they have enough flexibility and can easily get fixed in the cul-de-sac. The FZ-AG formulation showed the highest tensile strength because it was made from the crosslinked nanofibers with a higher amount of PVA. Tensile strength of the nanofibers was in the range of 3.124 ± 0.521 - 9.312 ± 0.104 MPa. Tensile strength of the CS fibers was improved as a result of mixing of spinning solutions with hydrophilic polymers, PVA, and PVP. Tensile strength was found to be higher in the FZ-AG and FZ-PG formulations compared to the FZ-A and FZ-P formulations, because of formation of inter-fiber bonding of nanofibers happening greatly at the intersection surface and making a rigid web of inter-fiber bonds, which could enhance mechanical properties of the crosslinked fibers compared to the non-crosslinked ones.^34,35^ Degree of swelling has a major effect on drug release from the nanofibers. The degree of swelling was found to be lower for the FZ-PG and FZ-AG formulations compared to the FZ-P and FZ-A formulations. Crosslinking of CS and PVA after exposure to GA, which increased intermolecular forces resulted in a decrease in swelling rate and consequently, slower rate of drug delivery.^35^ Moisture uptake and loss studies were conducted on the formulations. The results revealed that all the nanofibers had good physical stability at high humid and dry conditions. The prepared ophthalmic inserts indicated a surface pH between 5.99 ± 0.05 - 6.22 ± 0.01, which is suitable for ocular application and would not influence pH of tear fluid (Table 1).

**Table 1:** Physicochemical properties of the ocular inserts of azithromycin nanofibers (mean ± SD, n = 3)

**Formulation**	**Thickness (mm)**	**Folding endurance (times)**	**Tensile strength (MPa)**	**Moisture loss (%)**	**Moisture uptake (%)**	**Swelling (%)**
FZ-P	0.108 ± 0.012	153 ± 3	3.124 ± 0.521	1.40 ± 0.12	1.61 ± 0.05	125.3 ± 0.5
FZ-A	0. 115 ± 0.013	199 ± 2	4.136 ± 0.124	1.52 ± 0.01	1.56 ± 0.02	119.3 ± 0.2
FZ-PG	0.121 ± 0.002	164 ± 7	7.120 ± 0.204	0.78 ± 0.01	1.41 ± 0.01	104.3 ± 0.9
FZ-AG	0.116 ± 0.023	204 ± 3	9.312 ± 0.104	0.46 ± 0.22	1.17 ± 0.01	87.3 ± 0.4

### FT-IR spectroscopy

**Figure 2:**
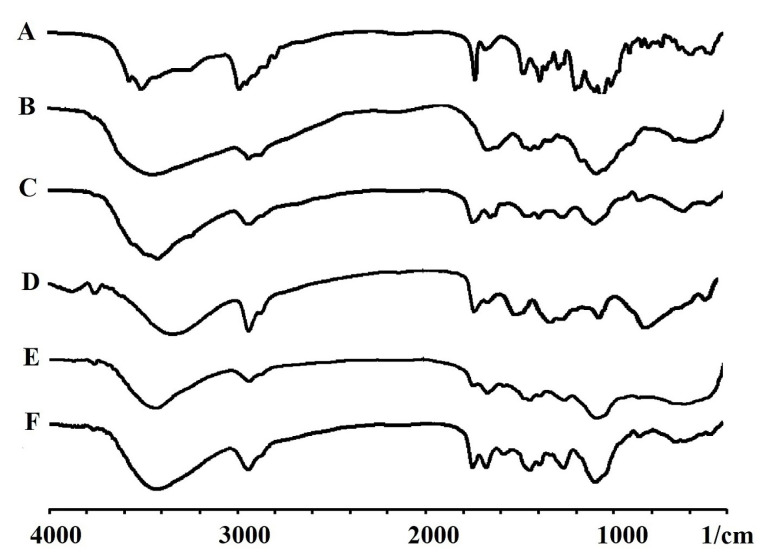
FTIR spectra obtained for azithromycin (A), chitosan (B), PVA (C), PVP (D), non-crosslinked (E), crosslinked (F) electrospun nanofibers.

Appearance of AZM characteristic peaks at nanofibers’ peaks conﬁrmed the presence of AZM inside nanofibers’ structure. Vibrational peak appeared at 848.68 cm^-1^ was related to C–H rocking mode of PVA, as shown in the spectra of nanofibers. ACH & CH asymmetric stretching vibration of PVA appeared at 2924 cm^-1^ was shifted to 2927 cm^-1^ in the AZM-loaded nanofibers.^36^ Strong absorption peaks at about 1292 cm^-1^, detected in PVP and nanofibers were assigned to the C–O bonding.^37^ The absorption bands at around 3412 and 3352 cm^-1^ were related to -OH and -NH stretching vibrations significantly shifted to a lower wave number by increasing PVA concentration in the mixtures, suggesting formation of hydrogen bonds between CS and PVA molecules. Chemical crosslinking of the CS/PVA was verified by detecting the peak located at 1566 cm^–1^ attributed to C-N bond. A peak at 1566 cm^−1^ was detected in the spectra of nanofibers crosslinked with GA, which could be related to crosslinking reaction of CS and GA (Figure 2).

Appearance of the characteristic peaks of AZM in nanofibers’ peaks conﬁrmed the presence of AZM inside the structure of nanofibers. Vibrational peak detected at 848.68 cm^-1^ was related to C–H rocking mode of PVA, which appeared in the spectra of nanofibers. ACH & CH asymmetric stretching vibration of PVA appeared at 2924 cm^-1^ was shifted to 2927 cm^-1^ in the AZM-loaded nanofibers.^36^ Strong absorption peaks at about 1292 cm^-1^, detected in the PVP and nanofibers were assigned to C–O bonding.^37^ The absorption bands at around 3412 and 3352 cm^-1^ were related to -OH and -NH stretching vibrations, significantly shifted to a lower wave number by increasing PVA concentration in the mixtures, suggesting formation of hydrogen bonds between CS and PVA molecules. Chemical crosslinking of CS/PVA was verified by detecting the peak located at 1566 cm^–1^ attributing to C-N bond. A peak at 1566 cm^−1^ appeared in the spectra of nanofibers crosslinked with GA, which could be related to crosslinking reaction of CS and GA (Figure 2).

### Characterization of nanofibers’ morphology

As can be seen in Figure 1, relatively fine, continuous, uniform, and randomly oriented nanofibers were obtained after electrospinning. Average diameter of the nanofibers was found to be 119.01 ± 29.77 nm for the non-crosslinked nanofibers (FZ-A formulation). The nanofibers undergone crosslinking process for 10 hours had an average diameter of 171.61 ± 39.40 nm (FZ-AG formulation). The results indicated that GA crosslinking slightly increases average diameter due to swelling of the nanofibers during the process.

### In vitro antimicrobial efficacy test

**Figure 3:**
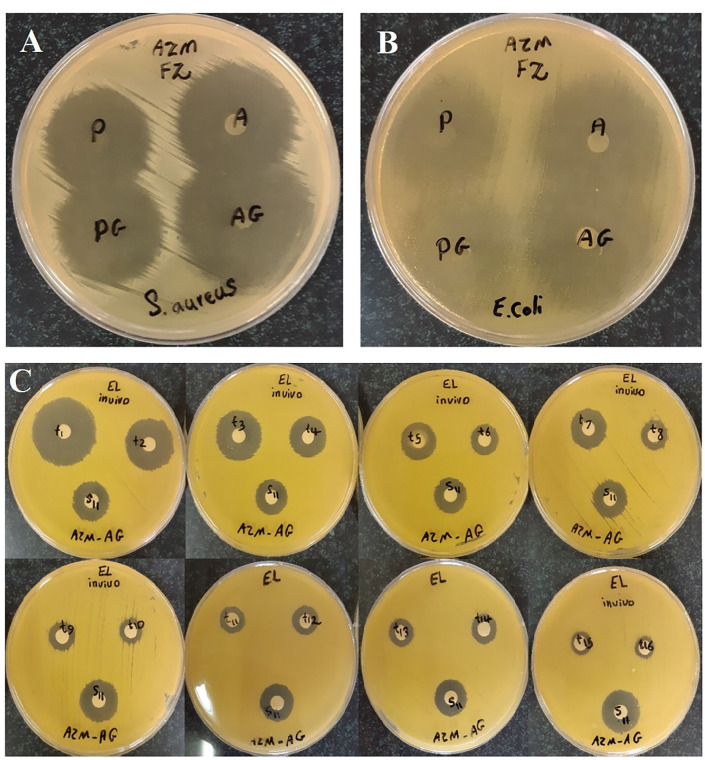
The *in vitro* antibacterial efficacy of non-crosslinked (FZ-A, FZ-P) and crosslinked (FZ-AG, FZ-PG) nanofibers against *S. aureus* (A) and *E. coli* (B). (C) The microbial assay plates cultured to quantify drug in samples collected from rabbits’ tear fluid following the administration of FZ-AG samples.”

Significantly clear inhibition zones were detected against both strains around all the AZM-loaded nanofibers. Slightly larger inhibition zones were found on the *S. aureus* and *E. coli* cultures for the crosslinked nanofibers compared to the non-crosslinked nanofibers (Figure 3) indicating that not only the crosslinked nanofibers preserved antibacterial activity but also, they showed significant higher activity compared to the non-crosslinked nanofibers. Reactivity of –NH2 groups in CS with GA was higher than quaternary ammonium groups. So, most of the groups with strong antimicrobial activity in CS did not react with GA and as a result, antibacterial efficacy was preserved.^38^

### Sterility test

The nanofibers were prepared under aseptic conditions and also were sterilized by UV radiation after preparation. Turbidity and growth of the microorganisms were observed in the positive controls, exhibiting that the culture media were appropriate for sterility test and there was no sign of growth in the negative controls confirming that the whole procedure of the test was sterile and aseptic condition was preserved. All the nanofiber formulations were sterile as there was no sign of microorganisms’ growth in the tested samples.

### Microbial assay

Microbiological assay is one of the most common methods for investigation of antibacterial agents including the macrolides.^39^ This method has been suggested as a drug quantification method by different pharmacopoeias^40,41^ and many other studies. The Japanese Ministry of Health and Welfare has previously carried out this method.^42^ Paper disk method has been introduced by many studies for quantification of antibacterial activity of the drugs. In a previous study, it was observed that the standard curve linearity was in the range of 0.003–2 mg/L and sensitivity of the assay was equal to 0.00390 mg/L for *Bacillus subtilis* regarding determination of AZM formulated into liposomes,^43^ also for determination of AZM in the solution, it was almost 50 μg/mL at the concentration range of 50-200 μg/mL.^44^ Among different types of commonly used microorganisms to quantify the antibiotics, *Micrococcus luteus* has been the best with an adequate sensitivity in detection of drug. Breier et al constructed a calibration curve for AZM with suitable linearity on a concentration range of 0.1–0.4 μg/mL in which, AZM concentration was determined in different pharmaceutical dosage forms by the cylinder–plate method and *Micrococcus luteus* was used as the testing organism.^45^ However, there is limited number of reports used this method to determine the macrolides incorporated into CS-PVA-PVP nanofibers, and none of which included release of AZM in tear fluid. In this paper, quantitative analysis of *in vivo* and *in vitro* release of AZM from different formulations was performed by the paper disk method. Diameters of growth inhibition zone of AZM standards (1000-0.122 µg/mL) were measured. There was a linear relationship between diameter of growth inhibition zone and log_10_ of drug concentrations for standards. Representative linear equation for *Micrococcus luteus* counts in analysis of AZM standards was as follows: y = 0.7308 xs+0.2109. The obtained correlation coefficient was equal to 0.9989, exhibiting good linearity. The coefficient of variation for individual standards ranged between 0.25 - 0.74%. As an example, Figure 3C displays the microbial assay plates cultured to quantify drug in samples collected from rabbits’ tear fluid following the administration of FZ-AG samples.

### In vitro study of drug release

**Figure 4:**
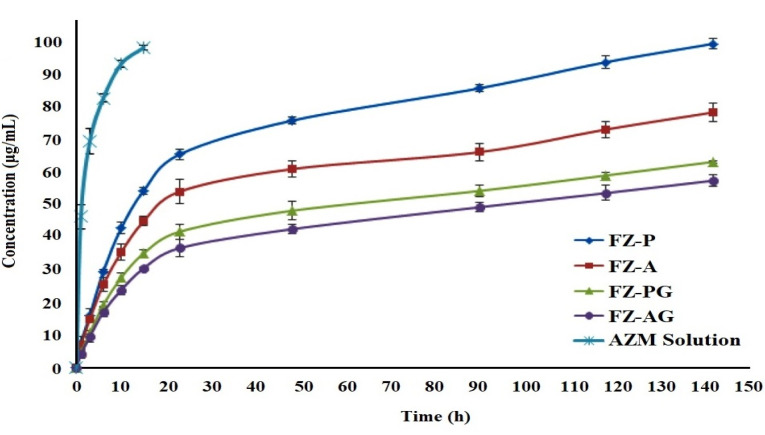
The *in vitro* release behavior of AZM from various formulations including FZ-P, FZ-A, FZ-PG, FZ-AG, and AZM solution in water.

Two-step release profile including an initial burst release step followed by the sustained release of drug was detected for all the formulations. After 23 hours, cumulative release percentage of the AZM was equal to 36.84 ± 2.81, 41.87 ± 2.37, 54.22 ± 3.83, and 65.67 ± 1.56% for FZ-AG, FZ-PG, FZ-A, and FZ-P formulations, respectively (Figure 4). The initial burst release of the nanofibers was due to accumulation of the drugs mostly on surface of the nanofibers. Different drug release rates for the formulations could result from different PVP/PVA blend ratios. As the PVP/PVA ratio was increased, swelling percentage of the nanofibers was enhanced, which led to more easily diffusion of the drug from the nanofibrous mats into the phosphate buffer.^46^ FZ-P formulation had a higher degree of swelling and as a result, higher drug release rate because of its higher PVP percentage compared to the FZ-A formulation (Table 1).

Due to GA crosslinking procedure, the CS/PVA formed a network chain structure with lower swelling percentage, which led to the decreased drug diffusion from the nanofibers and inhibited initial burst release of the drug.^35^ Drug release rate in the crosslinked nanofibers was relatively slower than that of the non-crosslinked nanofibers (Figure 4). The FZ-AG, FZ-PG, FZ-A, and FZ-P formulations showed nearly 57.59 ± 1.78, 63.39 ± 0.36, 78.60 ± 2.75, and 99.75 ± 1.47% of drug release after142 hours, respectively. All the formulations exhibited suitable prolonged release, so they were selected for ocular irritation test and *in vivo* studies.

### In vitro cytotoxicity test

**Figure 5:**
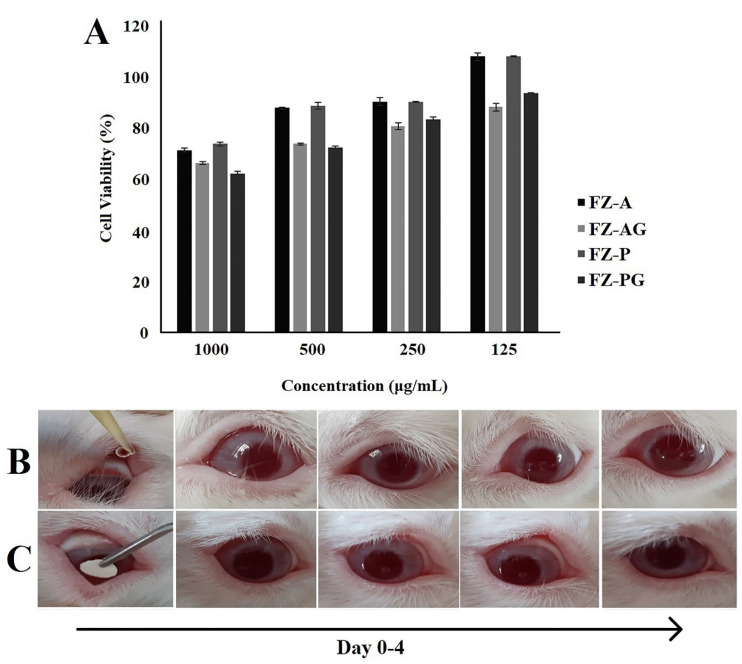
Cell viability measured by MTT assay (A) after treatment with different concentrations of nanofibrous formulations. Data are expressed as the mean ± SD of three separate experiments (n = 6). The appearance of rabbit eyes treated with 50 μL of PBS (B) and PBS + FZ-A nanofiber (C) through the irritation test using topical delivery acquired on day 0 to 4.

According to the results, an increase in concentration of the formulations led to the reduced cell viability (Figure 5). Nanofibers with low levels of cell cytotoxicity could be considered as safe systems for ophthalmic delivery of drugs. Similar results have been also reported on the L929 (mouse fibroblast) cells for the polycaprolactone nanofiber membranes as ophthalmic carriers. The crosslinked nanofibers showed slight reduction in viability of the cells compared to the non-crosslinked nanofibers. This may be due to trace amount of the residual GA on the nanofibers. Still, viability of the cells cultured with the crosslinked nanofibers was acceptable for ophthalmic application. Our results indicated that the AZM-loaded nanofibers had good biocompatibility, which made them appropriate ophthalmic carriers.

### Irritation test

No significant redness and continuous blinking of the eyes were observed (Figure 5). No ophthalmic damage or abnormality was detected in the cornea, iris, and conjunctiva after administration of PBS solution plus AZM-loaded nanofibers. It should be noted that very slight redness of the conjunctiva was observed but no chemosis was found after application of the crosslinked nanofibers.

### In vivo study of drug release

**Figure 6:**
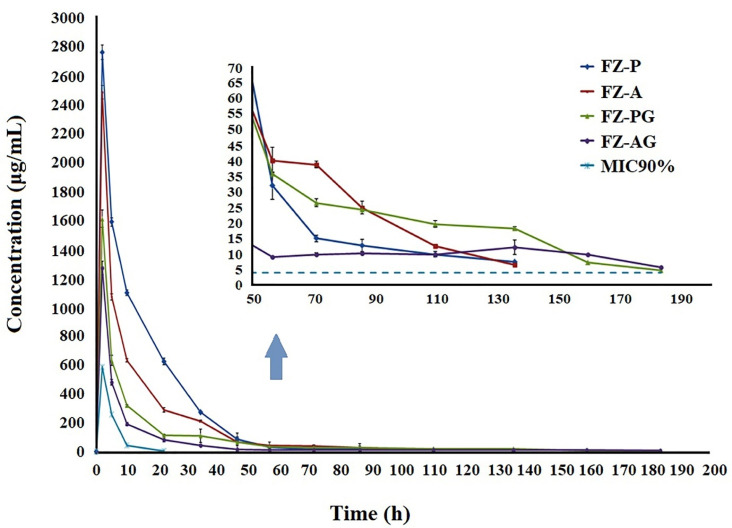
AZM concentration-time profile in tear fluid after application of sterilized AZM nanofibers with different formulations in rabbit eyes. FZ-P, FZ-A: AZM nanofibers before GA crosslinking, FZ-PG, FZ-AG: AZM nanofibers after GA crosslinking. Each point represents the mean ± SD.

The biodegradable nanofibers could be fixed in conjunctival sac of the rabbit eyes as ocular insert without the need for any invasive methods or surgery. The amount of released AZM in tear fluid from the nanofibers was measured by the microbial assay using the standard curve derived from standard AZM solutions, which is a non-invasive, inexpensive, and simple method (Figure 3). Figure 6 shows concentration–time curve of the AZM released in the rabbits’ tears from different nanofibers. The measured concentration of the AZM in tear from the solution was equal to 583.42 ± 54.32μg/mL after 2 hours. However, the measured concentrations were reported to reach below the limit of detection after 15 hours. The maximum concentration (Cmax) of FZ-AG and FZ-PG nanofibers measured after 2 hours of administration was equal to 1269.19 ± 94.58 and 1607.52 ± 59.94 μg/mL, respectively followed by a gradual release of the AZM in tear fluid, exhibiting the sustained release of drug within 184 h. Initial burst releases were observed for the non-crosslinked AZM-loaded nanofibers with Cmax of 2483.31 ± 46.3 and 2759.59 ± 51.45 μg/mL, respectively for FZ-A and FZ-P formulations,which were significantly higher compared to the crosslinked AZM-loaded nanofibers. A sustained release profile was observed following the burst phases (Figure 6). The lowest measured Cmax was related to the AZM solution due to rapid drainage, and reduced residence time.

The measured AUC_0–t_ of AZM for FZ-A, FZ-P, FZ-AG, FZ-PG formulations, and AZM solution was equal to 24504.38 ± 28.62, 35462.96 ± 154.76; 14688.09 ± 95.1, 15145.16 ± 150.32, and 2871.24 ± 143.03 μg h/mL, respectively (Table 2). Based on the obtained results, FZ-AG and FZ-PG formulations (crosslinked nanofibers) showed 5.11-fold and 5.27-fold increased AUC_0-t_ compared to the AZM solution, respectively. While, FZ-A and FZ-P formulations (non-crosslinked nanofibers) showed 8.53-fold and 12.49-fold increased AUC_0-t_ compared to the AZM solution, respectively. A significantly higher mean residence time (MRT) was measured for FZ-AG and FZ-PG formulations (crosslinked nanofibers) in comparison with the FZ-A and FZ-P formulations (non-crosslinked nanofibers) due to higher PVA concentration (3%) in the nanofibers besides GA crosslinking. The results suggested that formulation of AZM in the nanofibers may enhance pharmacokinetic behavior compared to the AZM solution.

**Table 2:** Pharmacokinetically obtained parameters from the *in vivo*study of the released drug in rabbit’s tear after drop instillation of 50 μL of AZM solution or AZM nanofibers with different formulations (mean ± SD, n = 6)

**Formulation**	**Cmax (μg/mL)**	**AUC**_0-t_ ** (μg h/mL)**	**MRT (h)**
FZ-A	2483.31 ± 46.30	24504.38 ± 28.62	17.97 ± 2.18
FZ-P	2759.59 ± 51.45	35462.96 ± 154.76	15.74 ± 0.11
FZ-AG	1269.19 ± 94.58	14688.09 ± 95.10	24.07 ± 1.28
FZ-PG	1607.52 ± 59.94	15145.16 ± 150.32	26.73 ± 2.11
AZM solution	583.42 ± 54.32	2871.24 ± 143.03	4.22 ± 0.20

In the previous studies, concentrations of AZM ranging from 0.05-4 μg/ml were considered as the minimum inhibitory concentration (MIC90) for common Gram-positive and Gram-negative organisms generally infecting the conjunctivitis. While, the AZM solution only achieved tear concentrations higher than the MIC90 for the first 10 hours, the non-crosslinked and crosslinked nanofibers were able to maintain concentrations up to the MIC range for 136 and 184 hours, respectively, which is required for treatment of ocular bacterial infections. As a result of this prolonged drug release, a less frequent administration of AZM is required and consequently, lower dosage of AZM is enough for achieving the desired therapeutic concentrations compared to the conventional AZM eye drop.

## Conclusion

In this study, electrospinning technique was successfully used to design and develop the CS/PVA-PVP nanofibers. The AZM was formulated in these delivery systems to provide a therapeutic amount of drug in the eye for a prolonged period. The prepared nanofibers were smooth and flexible along with sufficient hardness and weight uniformity.Studying *in vitro* drug release from the ophthalmic nanofibers indicated the sustained release of drug from all the formulations. The nanofibers were sterilized using UV radiation method and their sterility was confirmed by the sterility test. The results of MTT test carried out using the L929 fibroblast cells exhibited that neither of the formulations was significantly cytotoxic. The biodegradable nanofibers could be fixed in conjunctival sac of the rabbit’s eye as an ocular insert without the need for any invasive methods or surgery. There was no sign of significant inflammation or redness in the rabbits’ eyes after placement of the nanofibers. The drug release profile was evaluated by the microbial assay, as a non-invasive, inexpensive, and simple method and sampling was carried out using harmless sterile paper disks. The results obtained from* in vivo* study indicated the sustained release of drug in tear fluid for approximately 6-8 days from the AZM-loaded nanofibers, offering an enhancement in drug residence time on surface of cornea leading to an increased intraocular bioavailability in the eye. Our results revealed that the AZM-loaded nanofibers could be considered as suitable systems for ophthalmic delivery of AZM.

## Acknowledgments

The authors would like to acknowledge the Research Council of Kermanshah University of Medical Sciences (Grant number: 95522) for financial support of this work. Also, faithfully thank Rahesh Daru Novin knowledge-based company for cooperation in providing materials and equipment.

## Ethical Issues

The protocol for the use of animals in this study was in accordance with the Association for the Research in Vision and Ophthalmology (ARVO), which was approved by the Local Animal Ethics Committee of Kermanshah University of Medical Sciences (Approval No: IR.KUMS.REC.1396.568).

## Conflict of Interest

There was no conflict of interest declared.

## References

[1] Lang JC (1995). Ocular drug delivery conventional ocular formulations. Adv Drug Deliv Rev.

[2] Ghate D, Edelhauser HF (2006). Ocular drug delivery. Expert Opin Drug Deliv.

[3] Hughes PM, Olejnik O, Chang-Lin JE, Wilson CG (2005). Topical and systemic drug delivery to the posterior segments. Adv Drug Deliv Rev.

[4] Gaudana R, Ananthula HK, Parenky A, Mitra AK (2010). Ocular drug delivery. AAPS J.

[5] Fathi M, Barar J, Aghanejad A, Omidi Y (2015). Hydrogels for ocular drug delivery and tissue engineering. Bioimpacts.

[6] Mirzaeei S, Berenjian K, Khazaei R (2018). Preparation of the potential ocular inserts by electrospinning method to achieve the prolong release profile of triamcinolone acetonide. Adv Pharm Bull.

[7] Ghanbarzadeh S, Valizadeh H, Zakeri-Milani P (2013). Application of response surface methodology in development of sirolimus liposomes prepared by thin film hydration technique. Bioimpacts.

[8] AlMotwaa SM, Alkhatib MH, Alkreathy HM (2020). Incorporating ifosfamide into salvia oil-based nanoemulsion diminishes its nephrotoxicity in mice inoculated with tumor. Bioimpacts.

[9] Taghe S, Mirzaeei S (2019). Preparation and characterization of novel, mucoadhesive ofloxacin nanoparticles for ocular drug delivery. Braz J Pharm Sci.

[10] Taghe S, Mirzaeei S, Alany RG, Nokhodchi A (2020). Polymeric inserts containing Eudragit® L100 nanoparticle for improved ocular delivery of azithromycin. Biomedicines.

[11] Teo WE, Ramakrishna S (2006). A review on electrospinning design and nanofibre assemblies. Nanotechnology.

[12] Schiffman JD, Schauer CL (2008). A review: electrospinning of biopolymer nanofibers and their applications. Polym Rev.

[13] Sebe I, Szabó P, Kállai-Szabó B, Zelkó R (2015). Incorporating small molecules or biologics into nanofibers for optimized drug release: a review. Int J Pharm.

[14] Mehrandish S, Mirzaeei S (2021). A review on ocular novel drug delivery systems of antifungal drugs: functional evaluation and comparison of conventional and novel dosage forms. Adv Pharm Bull.

[15] Ludwig A (2005). The use of mucoadhesive polymers in ocular drug delivery. Adv Drug Deliv Rev.

[16] Patel A, Cholkar K, Agrahari V, Mitra AK (2013). Ocular drug delivery systems: An overview. World J Pharmacol.

[17] Calvo P, Remuñán-López C, Vila-Jato JL, Alonso MJ (1997). Novel hydrophilic chitosan-polyethylene oxide nanoparticles as protein carriers. J Appl Polym Sci.

[18] Ravi Kumar MN (2000). A review of chitin and chitosan applications. React Funct Polym.

[19] Rabea EI, Badawy MEI, Steurbaut W, Stevens CV (2009). In vitro assessment of N-(benzyl)chitosan derivatives against some plant pathogenic bacteria and fungi. Eur Polym J.

[20] Nagpal K, Singh SK, Mishra DN (2010). Chitosan nanoparticles: a promising system in novel drug delivery. Chem Pharm Bull (Tokyo).

[21] Ohkawa K, Cha D, Kim H, Nishida A, Yamamoto H (2004). Electrospinning of chitosan. Macromol Rapid Commun.

[22] Li L, Hsieh YL (2006). Chitosan bicomponent nanofibers and nanoporous fibers. Carbohydr Res.

[23] Duan B, Dong C, Yuan X, Yao K (2004). Electrospinning of chitosan solutions in acetic acid with poly(ethylene oxide). J Biomater Sci Polym Ed.

[24] Ignatova M, Manolova N, Rashkov I (2007). Novel antibacterial fibers of quaternized chitosan and poly(vinyl pyrrolidone) prepared by electrospinning. Eur Polym J.

[25] Yeh JT, Chen CL, Huang KS, Nien YH, Chen JL, Huang PZ (2006). Synthesis, characterization, and application of PVP/chitosan blended polymers. J Appl Polym Sci.

[26] Brinkley M (1992). A brief survey of methods for preparing protein conjugates with dyes, haptens, and cross-linking reagents. Bioconjug Chem.

[27] Tripathi S, Mehrotra GK, Dutta PK (2009). Physicochemical and bioactivity of cross-linked chitosan-PVA film for food packaging applications. Int J Biol Macromol.

[28] Retsema J, Girard A, Schelkly W, Manousos M, Anderson M, Bright G (1987). Spectrum and mode of action of azithromycin (CP-62,993), a new 15-membered-ring macrolide with improved potency against gram-negative organisms. Antimicrob Agents Chemother.

[29] Järvinen K, Järvinen T, Urtti A (1995). Ocular absorption following topical delivery. Adv Drug Deliv Rev.

[30] Zhou Y, Yang H, Liu X, Mao J, Gu S, Xu W (2013). Electrospinning of carboxyethyl chitosan/poly(vinyl alcohol)/silk fibroin nanoparticles for wound dressings. Int J Biol Macromol.

[31] Mishra DN, Gilhotra RM (2008). Design and characterization of bioadhesive in-situ gelling ocular inserts of gatifloxacin sesquihydrate. Daru.

[32] Liu X, Lin T, Fang J, Yao G, Zhao H, Dodson M (2010). In vivo wound healing and antibacterial performances of electrospun nanofibre membranes. J Biomed Mater Res A.

[33] Niu X, Long T, Chen Y (2016). Microbiological assay of a mixture of three lactic acid bacteria metabolites. Asian J Tradit Med.

[34] Destaye AG, Lin CK, Lee CK (2013). Glutaraldehyde vapor cross-linked nanofibrous PVA mat with in situ formed silver nanoparticles. ACS Appl Mater Interfaces.

[35] Yang D, Li Y, Nie J (2007). Preparation of gelatin/PVA nanofibers and their potential application in controlled release of drugs. Carbohydr Polym.

[36] Pal K, Banthia AK, Majumdar DK (2007). Preparation and characterization of polyvinyl alcohol-gelatin hydrogel membranes for biomedical applications. AAPS PharmSciTech.

[37] Taylor LS, Zografi G (1997). Spectroscopic characterization of interactions between PVP and indomethacin in amorphous molecular dispersions. Pharm Res.

[38] Yu Q, Song Y, Shi X, Xu C, Bin Y (2011). Preparation and properties of chitosan derivative/poly(vinyl alcohol) blend film crosslinked with glutaraldehyde. Carbohydr Polym.

[39] Horwitz W. Official Methods of Analysis of AOAC International. Volume I, Agricultural Chemicals, Contaminants, Drugs/Edited by William Horwitz. Gaithersburg, Maryland: AOAC International, 1997; 2010.

[40] USP. United States Pharmacopeia. Nineteenth Revision, United States Pharmacopeial; 2007.

[41] Chen WZ, Yang LH. Outline for British Pharmacopoeia 2011. Chinese Pharmaceutical Affairs; 2011. p. 9.

[42] Dubios M, Fluchard D, Sior E, Delahaut P (2001). Official methods for residual substances in livestock products official methods for residual substances in livestock products, 1994. J Chromatogr B Biomed Sci Appl.

[43] Solleti VS, Alhariri M, Halwani M, Omri A (2015). Antimicrobial properties of liposomal azithromycin for Pseudomonas infections in cystic fibrosis patients. J Antimicrob Chemother.

[44] Salgado HR, Roncari AF (2005). Microbiological assay for the determination of azithromycin in ophthalmic solutions. Yao Xue Xue Bao.

[45] Breier AR, Garcia CV, Oppe TP, Steppe M, Schapoval EE (2002). Microbiological assay for azithromycin in pharmaceutical formulations. J Pharm Biomed Anal.

[46] Mahmoudi Beram F, Koohmareh GA, Malekpour A (2019). Preparation and characterization of aqueous stable electro-spun nanofibers using polyvinyl alcohol/polyvinyl pyrrolidone/zeolite. Soft Materials.

